# Incorporation of an Intermediate Polyelectrolyte Layer for Improved Interfacial Polymerization on PAI Hollow Fiber Membranes

**DOI:** 10.3390/membranes13080741

**Published:** 2023-08-18

**Authors:** Maria A. Restrepo, Mehrdad Mohammadifakhr, Johannes Kamp, Krzysztof Trzaskus, Antoine J. B. Kemperman, Joris de Grooth, Hendrik D. W. Roesink, Hannah Roth, Matthias Wessling

**Affiliations:** 1 Chemical Process Engineering AVT.CVT, RWTH Aachen University, Forckenbeckstraße 51, 52074 Aachen, Germany; 2 MST-Membrane Science and Technology Cluster, Department of Science and Technology, Mesa+ Institute for Nanotechnology, University of Twente, P.O. Box 217, 7500 AE Enschede, The Netherlandsj.degrooth@utwente.nl (J.d.G.);; 3 Department of Research and Development, Aquaporin A/S, Nymøllevej 78, 2800 Kongens Lyngby, Denmark; 4 DWI-Leibniz-Institute for Interactive Materials, Forckenbeckstraße 50, 52074 Aachen, Germany

**Keywords:** interfacial polymerization, composite hollow fiber, polyamide-imide, chemistry in a spinneret, polyelectrolyte interlayer

## Abstract

In a single-step spinning process, we create a thin-walled, robust hollow fiber support made of Torlon^®^ polyamide-imide featuring an intermediate polyethyleneimine (PEI) lumen layer to facilitate the integration and covalent attachment of a dense selective layer. Subsequently, interfacial polymerization of m-phenylenediamine and trimesoyl chloride forms a dense selective polyamide (PA) layer on the inside of the hollow fiber. The resulting thin-film composite hollow fiber membranes show high NaCl rejections of around 96% with a pure water permeability of 1.2 LMH/bar. The high success rate of fabricating the thin-film composite hollow fiber membrane proves our hypothesis of a supporting effect of the intermediate PEI layer on separation layer formation. This work marks a step towards the development of a robust method for the large-scale manufacturing of thin-film composite hollow fiber membranes for reverse osmosis and nanofiltration.

## 1. Introduction

The core of reverse osmosis (RO), nanofiltration (NF), and forward osmosis (FO) membranes are separation layers rejecting ions [1]. These membranes consist of a thin selective layer on a porous support. The selective layer can be fabricated through different techniques, such as interfacial polymerization (IP), layer-by-layer assembly, or a conventional phase inversion process [2,3,4,5]. However, membranes comprising an interfacially polymerized polyamide (PA) layer offer the highest selectivity and water fluxes [1]. These types of membranes are also known as thin-film composite (TFC) membranes.

For RO, NF, and FO, hollow fibers with selective layers are highly desired since hollow fiber modules allow higher packing densities and backflushing, and are less prone to fouling [1,6,7]. In addition, due to the superior separation performance of PA, there is growing interest in producing hollow fibers with integrated PA selective layers. However, while IP on flat sheet membranes is already a well-established process and is, in fact, the standard for the fabrication of most commercial RO, NF, and FO membranes [8,9,10,11], IP on hollow fibers is not well established on a commercial scale [12].

Typically, TFC PA membranes are fabricated by soaking a porous support with an aqueous solution containing a functional amine, e.g., m-phenylenediamine (MPD). The support is then brought into contact with an organic solution containing a functional acyl chloride such as trimesoyl chloride (TMC). At the interface between the non-miscible aqueous and organic phases, the two monomers react to form a thin and dense selective polyamide (PA) layer [2]. The chemistry of both monomers used in the reaction plays a vital role in determining the properties of the resulting PA layer. For example, the reactivity and polarity of the functional groups on the amine and acyl chloride will affect the reaction rate and the extent of cross-linking [13]. Furthermore, the choice of support material can also influence the formation and properties of the PA layer. For instance, factors such as porosity and surface chemistry can affect the diffusion rate of monomers from the aqueous phase, which may impact the formation and adhesion of the PA layer [14,15].

However, all along, coating via IP on hollow fiber membranes suffers from the difficulty of achieving reproducible results and therefore lacks a high success rate in larger-scale manufacturing [15,16,17,18]. One of the main challenges in gaining reproducible TFC hollow fiber membranes is the uneven distribution of the monomer and the formation of droplets during the coating process. This may lead to defect formation, resulting in delamination and low selectivity. In flat sheet supports, the liquid–liquid interface is more accessible and can, for example, be controlled by removing the excess MPD solution with a rubber roller.

Some researchers have addressed the difficulty in achieving TFC hollow fibers by instead directly generating a selective layer during support fabrication. This approach, also known as “chemistry in a spinneret”, combines the formation of the porous support via phase inversion and a simultaneously occurring covalent or ionic cross-linking reaction on the membrane surface. As a result, TFC hollow fiber membranes evolve in a single step and prove to have well-integrated stable separation layers [19,20,21,22,23,24,25]. However, these methods have primarily yielded membranes with nanofiltration properties as the selective layer is primarily formed by the deposition of a charged polyelectrolyte layer.

Instead, a second approach consisting of integrating an intermediate layer on the hollow fiber support has been shown to improve the quality of a subsequent IP coating. Various studies report the coating of hollow fibers with intermediate layers and subsequently performing IP on said fibers. These layers comprised polyelectrolyte multilayers, polydopamine, or metal–-organic frameworks, among others. It was observed that, through the surface modification, the performance of the TFC membranes substantially increased [17,26,27,28]. Therefore, it might be argued that a defect-free PA layer can form by priming the surface with more favorable IP characteristics, leading to increased performance. Some groups have even explored the possibility of adding functional groups to the support layer, as it may further promote the adhesion of the PA layer via covalent bonding [29,30,31,32].

Recently, the chemistry in a spinneret approach has been used to create an intermediate layer that serves as a support for IP [18] or as a layer that subsequently cross-links itself [33]. Mohammadifakhr et al. [18] reached NaCl rejections of around 90% and reasonable water fluxes. Furthermore, they observed that adding a polyethyleneimine (PEI) intermediate layer led to more reproducible results than other positively charged polyelectrolytes. This higher success rate of IP was attributed to the presence of amino groups in PEI, which could potentially interact with TMC during the IP.

We propose utilizing polyamide-imide (PAI), commercially known as Torlon^®^, as a promising support material in membrane fabrication. PAI offers exceptional mechanical and chemical properties [34], making it well-suited for various applications such as high-pressure processes like NF and RO, as well as chemically demanding tasks like organic solvent nanofiltration [35,36,37]. Previous research by Setiawan et al. demonstrated the viability of PAI as a membrane material for FO hollow fiber membranes [38]. In their study, they modified PAI fibers by immersing them in an aqueous solution containing PEI, which facilitated cross-linking with PAI at elevated temperatures. This resulted in the formation of a positively charged surface layer with NF-like properties. However, their fibers only achieved 49% NaCl rejection, significantly lower than the rejection achieved with a thin selective PA layer.

**Figure 1:**
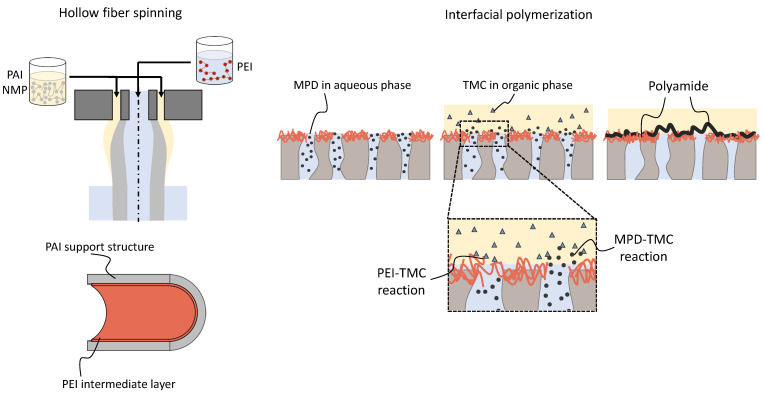
Schematic of the polyamide-imide (PAI) hollow fabrication with an incorporated polyethylenimine (PEI) intermediate layer and the subsequent interfacial polymerization (IP) of m-phenylenediamine (MPD) and trimesoyl chloride (TMC) performed in this work. On the left, a schematic representation of the dry–wet jet hollow fiber spinning, where the polymer solution containing PAI and the bore solution containing PEI are used. During the spinning process, PEI adsorbs to the inner surface of the hollow fiber, forming a composite membrane. On the right side, the figure depicts the subsequent modification using the IP of MPD and TMC. Both MPD and PEI react with TMC, enabling the covalent attachment of the newly formed polyamide layer to the support of the hollow fiber. This process results in the formation of defect-free thin-film composite hollow fiber membranes.

In this work, we evaluate the IP of PA on newly developed hollow fiber supports made of PAI with an intermediate PEI layer. First, we describe the hollow fiber fabrication process. Here, we used a dope solution containing PAI and a bore fluid containing PEI, as presented in Figure 1. We propose that the PAI/PEI fibers offer a well-integrated intermediate layer for the subsequent coating with an IP PA layer leading to a high success rate. MPD and TMC are used for the IP reaction. Next, the effect of the intermediate PEI layer is investigated by varying the MPD/TMC concentrations in the IP. We hypothesize that PEI takes part in the IP reaction and competes with MPD for the TMC resources (see Figure 1). All coated modules are then evaluated regarding their NaCl rejection and water permeability.

## 2. Materials and Methods

### 2.1. Materials

For the hollow fiber fabrication, polyamide-imide (PAI) Torlon^®^ 4000 TF was kindly received from Solvay Advanced Polymers (Brussels, Belgium). 1-Methyl-2-pyrrolidinone (NMP, 99%) was purchased from Acros Organics (Geel, Belgium). Branched polyethyleneimine (PEI) with a molecular weight ($M_{w}$) of ∼750 kDa ( 50% in H${}_{2}$O) and PEI with a $M_{w}$ of 25 kDa, ethylene glycol (EG), and glycerol (85–87%) were purchased from Sigma-Aldrich (St. Louis, MO, USA). For the IP and membrane modification, trimesoyl chloride (TMC) (>98%), and m-phenylenediamine (MPD) (>98%) were purchased from Sigma-Aldrich and were used without further purification. The isoparaffinic hydrocarbon Isopar E was used as organic solvent for TMC in the IP and was supplied by Brenntag A/S (Vejle, Denmark). Salts used for low-pressure reverse osmosis and FO measurements, NaCl from AzkoNobel A/S (Mariager, Denmark), and MgSO${}_{4}\;\cdot \;7$H${}_{2}$O from Tab-Sol (Straszecin, Poland), were food-grade quality.

### 2.2. Fabrication of Polyamide-Imide Hollow Fibers with an Intermediate Polyethylenimine Layer

The dope solution was prepared by adding 15 wt% PAI, 16 wt% EG, and 69 wt% NMP to a glass bottle and placing it on a roller bench until all components were fully dissolved. Then, the polymer solution was transferred to the container of the spinning machine, where it was degassed for 24 h. Similarly, bore solutions were prepared by adding the different components to glass bottles, as listed in Table 1, and mixing the solutions on a roller bench. Prior to the preparation of the dope solution, the PAI base polymer underwent drying in an oven at 100 ${}^{\circ }$C for 24 h. The fibers were fabricated using a dry-jet wet phase inversion spinning process. Here, the polymer solution is extruded through a spinneret along with the bore solution. Upon contact with the bore solution, the hollow fibers initiate phase inversion and are partly coagulated in the air gap. Then, the membranes fall through a coagulation bath where they fully solidify. Finally, the fibers pass through a rinsing bath and are collected by a winding wheel.

A total of three types of fibers were spun, each with a different bore solution as shown in Table 1. For each bore solution, different spinning parameters were employed, as indicated in Table 2. The first batch of fibers, referred to as “unmodified” fibers, was fabricated using bore solution B1. The other two fibers were fabricated with bore solutions containing 25 kDa PEI (B2) and 750 kDa PEI (B3), and are referred to as 25k and 750k, respectively.

After fabrication, the fibers were rinsed with deionized (DI) water for 72 h to remove residual NMP. Then, the fibers were immersed in a 30 wt% glycerol–water solution for 24 h to prevent pore collapse during the drying process. Lastly, the fibers were hung and air-dried at room temperature until further use.

### 2.3. Module Design

Four-end membrane modules consisted of polyethylene tubing (10 mm outer diameter) connected with two T-shaped Festo QS push-in fittings. Modules contain one or five fibers.

Fibers were fixed in place with a high viscosity two-component epoxy glue (Bison International B.V., Goes, The Netherlands). The effective length of the modules was 16 cm. The effective membrane area varies depending on the fiber type that was used. For membranes modified by MPD/TMC IP, membrane modules with 5 fibers each were prepared. For membranes modified using solely TMC, membrane modules with single fibers were prepared.

### 2.4. Membrane Modification via TMC Soaking

In order to assess the level of PEI adsorption on the inner surface of the hollow fiber and its reactivity with TMC, fabricated hollow fibers were immersed in a TMC solution. Modules containing a single hollow fiber were employed. The membranes were not pre-treated prior to the coating procedure, meaning that no water permeation was conducted to remove the glycerol from the porous structure. First, the membrane module was filled and soaked in DI water for 10 min. Afterwards, the module was fixed vertically and dried for 1 min with compressed air set at 0.5 L/min. Then, a TMC solution (0.5 wt% in Isopar E) was pumped through the fibers with a continuous flow of 2 mL/min for 2 min. The modules were then dried with compressed air for 30 s. Directly after the drying step, the modules were put in an air oven at 70 ${}^{\circ }$C for 10 min. Finally, modules were flushed with DI water. When not used, the modules were stored in the fridge in DI water at 7 ${}^{\circ }$C.

At least two membrane modules for each support type were prepared for membranes modified by only soaking them in an organic solution containing TMC.

### 2.5. Interfacial Polymerization

Five hollow fibers were present in the modules for IP modification. The dry modules were fixed vertically and soaked in MPD solutions of different concentrations (1.5, 2.5 and 3.5 wt%). A syringe pump created the continuous flow of 10 mL/min for 2 min. Afterwards, the modules were set horizontally and let to rest for 10 min to allow the solution to soak the pores completely. After 10 min, the pump tubing and the excess MPD solution were removed, and the drying tubing was connected at the top. The modules were dried in a vertical position for 1 min using compressed air and an airflow set to 1.6 L/min. The modules were fixed again vertically and connected to the TMC tubing. The TMC solution (0.1, and 0.2 wt% in Isopar E) was pumped at 10 mL/min with a syringe pump for 15 s. After 45 s without any flow, the pump started again and the solution flowed for 15 s, followed by another 45 s resting period. The module was dried again from the top in a vertical position with compressed air at 1.6 L/min for 30 s. Subsequently, the module was put in an oven at 70 ${}^{\circ }$C for 10 min. Finally, to remove residual MPD and TMC, the modules were flushed overnight with DI water at 2 bar in a cross-flow setting. When not used, modules were stored in a fridge and kept in DI water at 5 ${}^{\circ }$C.

For membranes modified with by the MPD/TMC IP, at least two modules of each fiber type were prepared for each coating.

### 2.6. Membrane Characterization

#### 2.6.1. Porosity

The fibers underwent an initial rinsing step for 20 min in an inside-out configuration. Subsequently, they were placed in a vacuum oven at a temperature of 30 ${}^{\circ }$C for a minimum of 4 h prior to testing. The porosity of the fibers was determined using the known density value of polyamide-imide. Before measuring the mass of the sample, the sample was placed in a vacuum oven for at least 1 h. The porosity was calculated using Equation (1).

(1) $$\varepsilon =\frac{V-\frac{m}{\rho }}{V}$$

where *m* represents the mass of the sample (g), ρ is the density of PAI (g·cm${}^{-3}$), and *V* denotes the volume of the fiber (cm${}^{-3}$). The density value used for the polyamide-imide in the calculation was 1.4 g· cm${}^{-3}$ [39]. To ensure accuracy, triplicate tests were conducted for each type of fiber. The porosity measurement was performed solely on the unmodified, 25k, and 750k fibers without any form of coating.

#### 2.6.2. Burst Pressure

The burst pressure of individual fibers was evaluated using a custom setup (Demcon Convergence, Enschede, The Netherlands). The setup applies glycerol to the hollow fiber, gradually increasing the pressure at a rate of 0.5 bar · s-1 until the fiber fails. The maximum pressure at which failure occurs is recorded as the burst pressure. The tests were conducted using a single fiber membrane module under ambient room temperature conditions.

#### 2.6.3. Microscopy Analysis

To analyze the morphology of the fibers prior to any coating, scanning electron microscopy (SEM) imaging was used. The fibers were first frozen using liquid nitrogen and then fractured to create cross-section samples. A thin layer of chromium with a thickness of 5 nm was sputter-coated onto the surface of the samples using a Quorum Q150T ES sputter coater (Quorum, Laughton, UK). SEM imaging was conducted using a JEOL JSM-6010LA instrument (JEOL, Tokyo, Japan).

To analyze the morphology of the PA layer, field emission scanning electron microscopy (FESEM) imaging was used. For the surface morphology, the fibers were cut lengthwise using a sharp razor blade. To obtain the cross-section of the PA layer, fibers were soaked in ethanol for 3 min and then frozen and fractured in liquid nitrogen. FESEM imaging was realized using a Hitachi S-4800 (Hitachi, Tokyo, Japan).

#### 2.6.4. Zeta Potential

The zeta potential of the fibers was determined using an electrokinetic SurPASS analyzer (Anton Paar, Graz Austria) at various pH values (3–11) in the presence of a 5 mM KCl electrolyte solution.

For the measurement, three individual fibers from each type were individually placed in tubings measuring 7 cm in length to enable triplicate measurements. Two solutions of 0.1 M HCl and 0.1 M NaOH were used to automatically adjust the pH. The zeta potential was calculated using Equation (2).

(2) $$\zeta =\frac{dI}{dP}\;\cdot \;\frac{\eta }{\varepsilon \;\varepsilon_{0}}\frac{L_{s}}{A_{s}}$$

where *I* is the streaming current (V), *P* is the pressure (Pa), η is the dynamic viscosity of the electrolyte solution (Pa·s), ε is the dielectric permittivity of water, $\varepsilon_{0}$ is the dielectric permittivity in vacuum (F·m${}^{-1}$), $L_{s}$ is the channel length (m), and $A_{s}$ is the cross-sectional area of the streaming channel (m${}^{2}$).

#### 2.6.5. Salt Rejection and Water Permeability

Rejection experiments were performed in a cross-flow set-up at a transmembrane pressure (TMP) of 2 bar. The cross-flow rate at each fiber was set at 5 mL/min. For salt rejection experiments, salt solutions containing 0.5 g/L ( 8.6 mmol/L) NaCl dissolved in DI water or 0.5 g/L (4.2 mmol/L) MgSO${}_{4}$ dissolved in DI water were used. Salt rejection was calculated with Equation (3).

(3) $$R=1-\frac{\sigma_{P}}{\sigma_{F}}$$

In this equation, *R* is the salt rejection (%), and $\sigma_{P}$ and $\sigma_{F}$ denote the measured conductivity of the permeate and the feed, respectively, (µS·cm${}^{-1}$). The water permeability was determined from Equation (4).

(4) $$P=\frac{V}{S_{A}\;\cdot \;\Delta t\;\cdot \;\left(\Delta p-\Delta \pi \right)}$$

In this equation, *P* is the water permeability (L·m${}^{-2}\;\cdot \;$h${}^{-1}\;\cdot \;$bar${}^{-1}$, LMH/bar), *V* the the collected volume (L), $S_{A}$ the effective membrane area (m${}^{2}$), Δt the recorded time (h), Δp the measured TMP (bar), and Δπ the osmotic pressure difference across the membrane (bar) [40]. The latter was calculated using the Van ’t Hoff Equation [41]:

(5) π=ciRT

where *c* is the concentration of the salt in the solution (mol·L), *i* the the Van’t Hoff factor of the specific salt, *R* the universal gas constant (J·K${}^{-1}\;\cdot \;$mol${}^{-1}$), and *T* the temperature (K). To calculate the osmotic pressure, only the concentration of the bulk solution was taken into account. The osmotic pressure generated from concentration polarization was calculated to be much lower than the osmotic pressure generated from the bulk solution and was therefore neglected.

After the measurements were finished, the membrane modules were flushed with DI water overnight at 2 bar.

## 3. Results and Discussion

In the following section, we present the interfacial polymerization (IP) of MPD and TMC on the novel hollow fibers. First, the characteristics of three PAI hollow fiber membrane batches are shown. Two of the batches are modified with a PEI lumen layer, which was deposited directly during the spinning of the fibers. Afterwards, these batches are used as support for further cross-linking. TMC cross-links with the PEI surface layer. IP with MPD and TMC creates a thin separate polyamide layer on the inside of the fibers. Last, we present the results for fibers modified with the IP of MPD and TMC.

For membrane characterization, salt retention and permeability were measured. As for nomenclature, we refer to fibers spun with 25 kDa PEI as 25k, and fibers spun with 750 kDa PEI as 750k.

### 3.1. Intrinsic Characteristics of the Hollow Fiber Support

**Figure 2:**
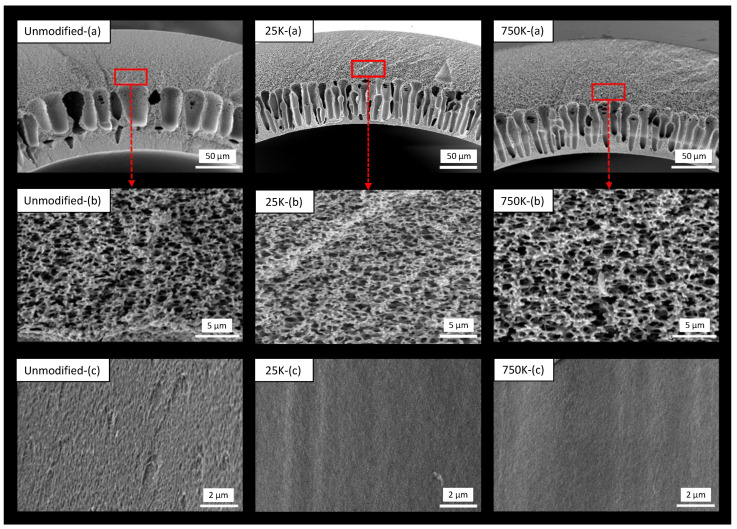
SEM images of unmodified, 25k, and 750k fibers; (**a**) cross-section, (**b**) morphology of the middle part, (**c**) lumen surface.

This section discusses the intrinsic properties of the fabricated hollow fiber supports. Hollow fiber dimensions, burst pressure, and pure water permeability are listed in Table 3. Despite their relatively thin wall of a thickness of around 0.15 mm, the spun fibers still showed a high burst pressure of around 14 bar on average, which can be explained by the high tensile strength of the polyamide-imide. Furthermore, the variation in the molecular weight of PEI had no discernible effect on the burst pressure. Moreover, the fibers displayed a porosity of >73%, indicating an open structure, as confirmed by SEM images showcasing the interconnection between the pores (see Figure 2).

SEM images of unmodified, 25k, and 750k fibers are shown in Figure 2. A morphology of combined finger-like and spongy structures is observed from the cross-section images of the three fibers. However, we observe some differences between the structures of the unmodified and the 25k and 750k fibers. For the unmodified fibers, macrovoids are present in the middle of the cross-section, while the sponge-like structure is on both the lumen and the shell side. In contrast, for the 25k and 750k supports, we observe the macrovoid formation directly at the lumen of the fibers with the sponge-like structure only at the shell. We owe this difference to the hindered solvent exchange due to the PEI layer formation; hence, the macrovoid formation shifts towards the lumen surface. The three fiber types have an open structure, highly porous in the middle of the fibers. A visible difference in the inner surface of the unmodified surface and the 25k and 750k can be observed, while the unmodified fibers exhibit a rough and uneven surface, the inner surface of both 25k and 750k is smooth and uniform.

**Figure 3:**
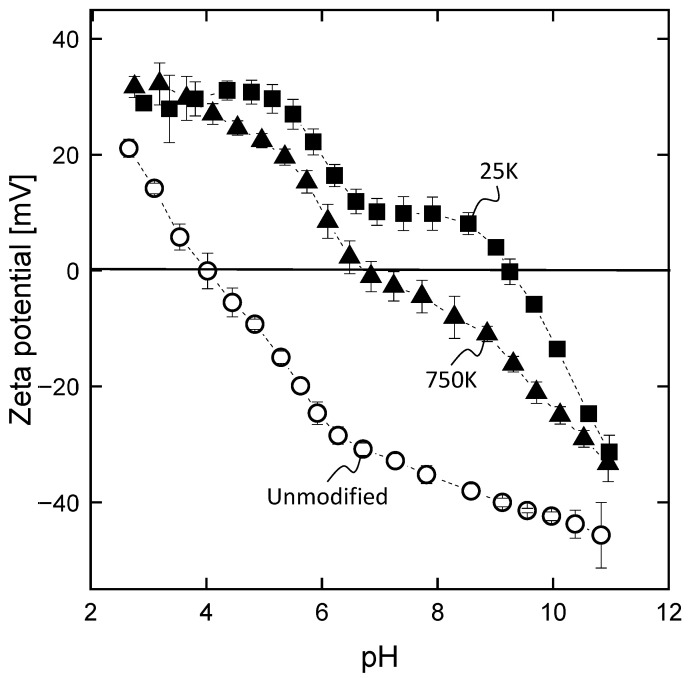
Zeta potential of the inner surface of hollow fiber membranes without (unmodified) and with (25K, 750K) intermediate PEI layers on the lumen of the fibers as a function of pH, *n* = 3.

The results of zeta potential measurements of the two different PAI/PEI fibers and the reference PAI (unmodified) fiber are compared in Figure 3. The overall stronger positive zeta potential proves the incorporation of the PEI on the surface compared to the reference. Furthermore, two plateau steps are observable for the PAI/PEI fibers, representing the two protonation stages of PEI’s primary and secondary amines. At a given pH, the zeta potential of the fiber spun with 25 kDa is more positive compared to fiber spun with 750 kDa PEI. The more positive zeta potential observed in the 25k membranes suggests a higher incorporation of amino groups. We theorize this difference is due to the variation in chain lengths of the polyelectrolytes used in the fabrication process. The smaller molecular weight of 25 kDa PEI enables greater mobility, facilitating deeper penetration of PEI into the PAI matrix during the phase inversion process. In contrast, the larger 750 kDa PEI diffuses more slowly, leading to its immobilization primarily on the outermost surface, which may be more susceptible to washing away during subsequent steps.

### 3.2. Membrane Modification through TMC Cross-Linking

**Figure 4:**
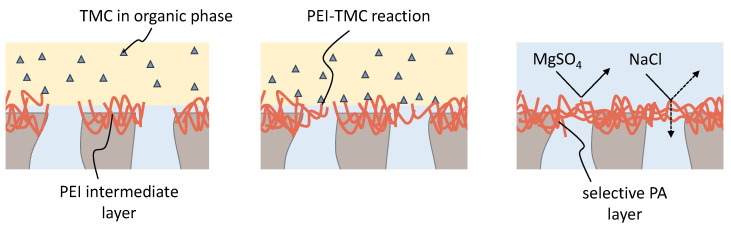
Schematic representation of the cross-linking of the intermediate layer upon contact with an organic solution containing TMC. The reaction of PEI and TMC leads to the formation of a dense selective layer with nanofiltration properties.

Before conducting the actual IP, cross-linking the PEI layer with TMC serves as an additional reference membrane. In the following section, we discuss the ability of the surface PEI layer to cross-link with TMC. The cross-linking is schematically shown in Figure 4.

**Figure 5:**
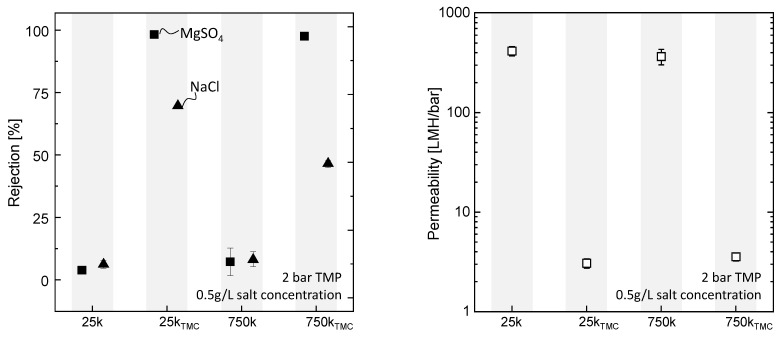
Performance of 25k and 750k hollow fibers before and after treatment with TMC, *n* = 2. On the left: salt rejection of MgSO${}_{4}$ (□) and NaCl (△). On the right: permeability measured during MgSO${}_{4}$ salt rejection experiments.

To investigate the effect of TMC on the 25k and 750k PAI/PEI fibers, we exposed the fibers to the TMC solution in a set of experiments. The resulting fibers are labeled as 25k${}_{TMC}$ and 750k${}_{TMC}$. We compare MgSO${}_{4}$ and NaCl rejections, as well as the permeability of the evolving fibers, with the unmodified 25k and 750k support fibers, given in Figure 5.

For the 25k and 750k, no salt rejection is measured. A cross-linking reaction of the PEI surface layer with solely TMC results in rejections of about 98% MgSO${}_{4}$ for both 25k${}_{TMC}$ and 750k${}_{TMC}$ membranes, and NaCl rejections of 68% and 44% for 25k${}_{TMC}$ and 750k${}_{TMC}$ fibers, respectively. Moreover, the permeability of the TMC-treated supports decreases due to the build-up of a dense selective separation layer.

The high rejection of the divalent ions of MgSO${}_{4}$ and moderate rejections for the monovalent ions of NaCl for both membranes are typical for nanofiltration membranes. Here, Donnan exclusion is superimposed with steric exclusion. The charge of the resulting membrane surface was not investigated. However, other studies report the formation of NF membranes through the IP of PEI and TMC. There, a positive surface charge is suggested [42,43,44]. On the other hand, negative charges can evolve due to the hydrolysis of unreacted acid chloride groups in TMC, forming hydroxyl groups [33]. We assume the higher NaCl rejections of the 25k${}_{TMC}$ fibers are due to the formation of a denser polymer network. This is in accordance with the stronger positive zeta potential of the 25k fibers indicating a higher amount of PEI amino groups that can react with TMC.

In a recent work by Gao et al. [33], NF membranes were fabricated following a similar procedure described here. However, in their work, they modified their fibers’ properties at even shorter TMC exposure periods (1 min). This suggests that even a shorter reaction time leads to the formation of a dense separation layer, limiting further diffusion of TMC to the underlying support. This diffusion barrier might impact the reaction when MPD and TMC are used together for the IP. In this case, PEI competes with MPD for the TMC resources, and the reaction between PEI and TMC might lead to a premature formation of a selective layer, hindering the diffusion of MPD. Although, in theory, steric effects in hyperbranched PEI could lead to a slower reaction rate compared to MPD [45].

### 3.3. Membrane Modification through Interfacial Polymerization of MPD and TMC

In the following section, we investigate the suitability of the PAI/PEI fibers as support for IP using MPD and TMC. Changes in the used MPD and TMC concentrations reveal their influence on the PA layer formation. Both types of fibers (25k, 750k) were coated following the protocol described in Section 2, with varying MPD and TMC concentrations (MPD: 1.5, 2.5 and 3.5 wt%; TMC: 0.1 and 0.2 wt%). We characterize the resulting membranes by their NaCl rejection and water permeability. Finally, the morphologies of the PA layers are discussed by comparing FESEM images.

**Figure 6:**
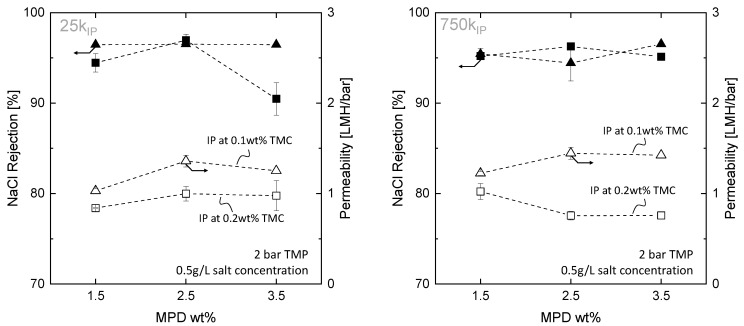
NaCl rejection and permeability of membrane modules prepared with 25k supports (**left**) and 750k supports (**right**) coated with different MPD concentrations (1.5, 2.5 and 3.5 wt%) and 0.1 wt% TMC (triangular makers), and 0.2 wt% TMC (square marker), *n* = 2.

The rejection of NaCl and the water permeability for membranes prepared with 25k and 750k supports and different concentrations of MPD and TMC are depicted in Figure 6. These modified fibers will be referred as 25k${}_{IP}$ and 750k${}_{IP}$. We observed that 25k${}_{IP}$ membranes prepared with the lower TMC concentration ( 0.1 wt%) achieved high rejections for all three MPD concentrations. However, when the TMC concentration was increased to 0.2 wt% TMC, rejection decreased for membranes prepared with 1.5 and 3.5 wt% MPD. Moreover, we observed a general increase in permeability with increasing MPD concentration from 1.5 to 2.5 wt%, while further increasing MPD concentration to 3.5 wt% yielded no significant changes. However, membranes prepared with 0.2 wt% yielded less permeable overall. In contrast, for 750k${}_{IP}$ membranes, the effect of MPD and TMC is less evident in the salt rejection, as the latter always remains above 95 wt% for all MPD and TMC concentrations. Furthermore, while permeability increases with increasing MPD concentration at 0.1 wt% TMC, the opposite is observed at 0.2 wt% TMC.

Overall, the results suggest that the effect of MPD and TMC on the rejection and permeability of the 25k and 750k membranes is complex and depends on the specific conditions. The following section discusses the individual effects of MPD and TMC in the IP and their possible consequences on membrane performance.

#### 3.3.1. Effect of MPD Concentration

**Figure 7:**
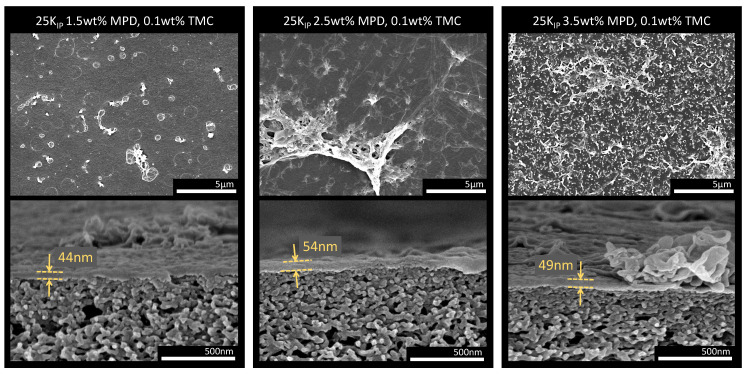
FESEM images of the lumen surface and cross-section of TFC hollow fiber membranes prepared with 25k supports and different concentrations of MPD and TMC. (**Left**) 1.5 wt% MPD and 0.1 wt% TMC. (**Middle**) 2.5 wt% MPD and 0.1 wt% TMC. (**Right**) 3.5 wt% MPD and 0.1 wt% TMC.

To better understand the effect of the MPD concentration, we compare the obtained rejections and permeabilities to the evolving surface morphology of the membranes depicted in Figure 7. The morphology of the 25k${}_{IP}$ membranes prepared with various concentrations of MPD and TMC are presented in Figure 7. Membranes coated with 1.5 wt% MPD and 0.1 wt% TMC (a) depict a rather smooth surface morphology. By increasing the concentration of MPD to 2.5 wt% and keeping the TMC concentration at 0.1 wt%, the surface roughness increases because of what we assume are protruding thin PA layers folding on top of the main layer. This is also accompanied by the formation of looser PA aggregates. However, the increase in roughness is even more evident at a concentration of 3.5 wt% MPD. Moreover, the cross-section of the fiber revealed that the PA layer did not exhibit the typical “ridge-and-valley” structure found in most TFC PA membranes. Instead, we observed individual nodular or ear-like protuberances emerging in combination with some larger agglomerations of PA. We attribute the overall increase in surface roughness to the greater driving force for diffusion resulting from the higher MPD concentration. The increased movement of MPD molecules towards the organic phase extends the reaction beyond the interface, leading to the formation of a rougher surface. This phenomenon has been well-documented in existing literature [46,47,48,49,50,51,52,53].

Interestingly, all of the fibers present a very thin PA layer of around 50 nm thickness. However, the expected layer thickness for this range of MPD and TMC concentration is around 200–250 nm if a conventional support like polysulfone (PSF) is used [47,54,55]. In the case of PSF, the larger apparent thickness is due to void formation inside the PA layer, which at the same time is responsible for the classic “ridge-and-valley” structure. Furthermore, void-free layer formation is observed in PSF supports when very low MPD concentrations are used [47]. However, PSF does not chemically interact with the monomers in the IP reaction, as is the case with our PAI/PEI fibers. As demonstrated in the previous section, the intermediate layer can react with TMC and form a selective layer. The reaction of a “premature” dense layer can lead to limited MPD transport to the organic phase, explaining the apparent thin layer. In a study by Chiao et al. [45], it was observed that when PEI and MPD are competing monomers in the IP, the ridge-and-valley structure gradually decreases and eventually disappears with increasing PEI concentration. Hence, increasing PEI concentration led to the formation of a smooth and thin PA layer.

As mentioned previously, only a slight increase in roughness was observed by increasing the MPD concentration from 1.5 to 2.5 wt%, and a more significant increase at 3.5% MPD (see Supplementary Materials). While numerous studies have consistently established a correlation between increased surface roughness and enhanced water flux [50,51,52,53,56], we only observed a distinct increase in permeability between fibers coated with 1.5 and 2.5 wt% MPD. Surprisingly, despite the more pronounced increase in roughness between 2.5 and 3.5 wt%, no further increase in permeability was evident. Our theory suggests that at the lowest concentration of MPD, the formation of the PA layer may occur partially within the support pores, which explains the measured low permeability. However, as the MPD concentration increases, the reaction is shifted toward the lumen surface, resulting in increased permeability due to reduced PA formation inside the pores. At the highest MPD concentration ( 3.5 wt%), with the monomer more readily accessible to TMC, a more cross-linked PA layer can form, facilitated by the higher concentration of monomer units available to react with each other. Thus, an increase in layer density would effectively balance the gained surface area.

#### 3.3.2. Effect of TMC Concentration

As discussed previously, TMC also has a significant effect on membrane performance. In the case of 25k${}_{IP}$ fibers, by increasing the TMC concentration from 0.1 to 0.2 wt%, both rejection and permeability decrease. In contrast, for 750k${}_{IP}$ fibers, only a decrease in permeability is observed (Figure 6). These results differ significantly from results found in the literature with IP on chemically inert support structures. Several studies report that, for the concentration range of MPD and TMC used in this study, an increase in TMC concentration can lead to either an increase in water permeability, an increase in salt rejection, or an increase in both [46,47,57]. The increase in water permeability has been linked to a decrease in PA layer thickness [46,58], whereas an increase in salt rejection has been linked to an increase in PA density degree [46,47].

However, compared to results from the literature, the underlying support membrane does not chemically react with the IP monomers. In the case of the PAI/PEI fibers, not only does the support itself cross-link to form a dense PEI-TMC PA layer, this layer and the support itself can cross-link to the nascent MPD-TMC PA layer. Although the TMC and MPD concentrations are similar, the mechanisms and interactions with the support influence the reaction differently. This opens up the possibility for a simultaneous reaction between MPD-TMC and PEI-TMC during the IP. Furthermore, a reaction between TMC and the primary and secondary amino groups in PEI could tighten the PEI support layer during the IP, further limiting the diffusion of MPD into the organic phase. Although stability during operation might be provided by covalent bonding between PA and PEI through the cross-linking with TMC, e.g., stability against delamination, the same could present a limiting factor for the reaction between MPD and TMC.

**Figure 8:**
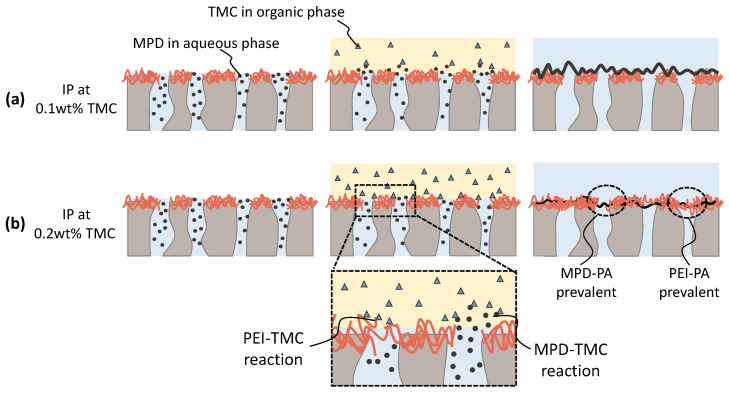
Schematic representation of the hypothesized interfacial polymerization (IP) of PAI support membranes with a PEI lumen layer coated with (**a**) 0.1 wt% TMC and (**b**) 0.2 wt% TMC. The competing reaction between PEI-TMC and MPD-TMC is presented. The polyamide (PA) layer formed through the reaction between PEI-TMC is less selective towards NaCl than the layer formed through MPD-TMC.

We propose a reaction mechanism between TMC and MDP in the presence of a PEI surface layer, which is illustrated in Figure 8. Here two scenarios are portrayed. The first scenario represents a low concentration of TMC (a). The low TMC concentration does not lead to the rapid formation of a cross-linked PEI layer. Therefore, the diffusion of MPD into the organic TMC phase is not as severely hindered, and a smooth but selective layer is formed. At this stage, the selective layer consists mainly of PA formed through the reaction of MPD and TMC, as reflected by the high rejections. A second scenario portrays a higher concentration of TMC (b). Due to the higher concentration of TMC, the reaction of PEI and TMC is more dominant than in the 0.1 wt% TMC case, reducing the diffusion of MPD into the organic TMC phase. As a result, a selective layer of PEI-TMC PA is formed at the surface of the hollow fiber, while a reaction of MPD and TMC would take place deeper inside the pores. Indeed, inside-pore PA formation has been reported to significantly reduce water permeance [59].

While support tightening from a PEI-TMC reaction combined with an in-pore MPD-TMC PA formation may explain a decrease in permeability, a reduction in rejection can be due to a combination of factors. In this study, we investigated the formation mechanisms of the PA layers, distinguishing between the PEI-TMC and MPD-TMC reaction systems, while the MPD-TMC reaction has been extensively studied, the superposition of different reaction routes in the PEI-MPD-TMC system is even more complex. Based on our findings, we hypothesize that the formation of the PEI-PA layer is more prevalent at higher TMC concentrations, hindering the diffusion of MPD and resulting in a less dense PA network, which may lead to lower rejection for 25k${}_{IP}$ fibers. However, we did not observe a decrease in rejection for 750k${}_{IP}$ fibers coated with 0.2 wt% TMC, possibly because 750k fibers have less available PEI amino groups on their surface and are less reactive towards TMC (see Section 3.1). Consequently, the impact of the PEI-TMC PA formation is stronger for the 25k fibers than the 750k supports. Although there is still a cross-linking of PEI, resulting in the overall decrease in permeability, the formation of the selective layer is primarily due to the reaction of MPD and TMC. Moreover, in the case of the 750k support, additional factors such as increased layer density may impact permeability as well. Indeed, these observations highlight the complexity of the PAI/PEI support with the MPD-TMC system, and further research is necessary to fully comprehend the reaction system and unravel the interplay between various parameters and their influence in membrane performance.

### 3.4. Success Rate of IP

**Figure 9:**
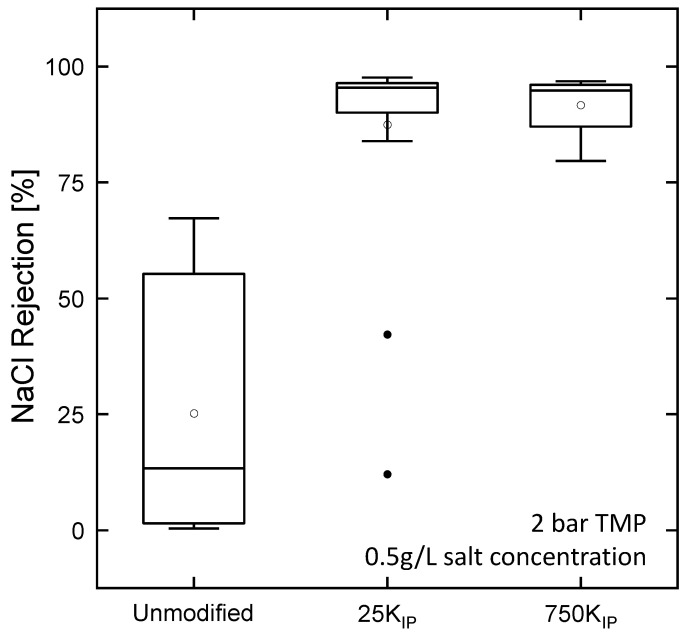
NaCl rejection of hollow fibers after IP coating of supports without a PEI intermediate layer (unmodified) and with a PEI intermediate layer (25k${}_{IP}$ and 750k${}_{IP}$). A total of *n* = 6 modules for the unmodified support were coated, and *n* = 21 modules of each 25k${}_{IP}$ and 750k${}_{IP}$. White markers represent the average rejection and black dots represent outlier samples.

IP is notably challenging to achieve on hollow fiber supports, as the interface is more difficult to control. Improper drying or excessive drying may lead to the formation of defects. Moreover, improper adhesion of the PA layer may lead to delamination. Figure 9 depicts the NaCl rejection of all membranes produced during this study. From the unmodified fibers (without PEI intermediate layer), none achieved a NaCl rejection > 80%. In comparison, most of the 25k and 750k supports achieved rejections higher than 80%, with a majority of the modules having rejections higher than 90%. Therefore, we attribute the high success rate of 25k${}_{IP}$ and 750k${}_{IP}$ membranes to the ability of TMC to interact with the primary amines in PEI during the IP. Hence, the PA layer can covalently bind to the support structure, resulting in a well-integrated PA layer, increasing layer stability, and preventing delamination. This is in accordance with previous studies, which attribute the high success rate of the IP on hollow fibers to the incorporation of a PEI intermediate layer through LBL deposition [17] or directly during the spinning process [18].

It is worth mentioning that the membranes produced in this study demonstrate water permeabilities of approximately 1 LMH/bar, falling within the reported range of RO membranes, which range from 0.9 to 3 LMH/bar [60,61,62]. On the other hand, the achieved salt rejection of approximately 95–96% may initially seem relatively low compared to the >99% achieved in commercial RO membranes. However, it is essential to consider the factors influencing salt flux across the membrane. The salt flux through the membrane is primarily determined by the permeability of salt in the polyamide layer and the salt concentration in the feed solution. In contrast, the water flux depends on the pure water permeability of the membrane and the difference between TMP and osmotic pressure.

In the present study, a TMP of 2 bar was utilized, which is low compared to pressures used in RO systems (>15 bar). Hence, increasing the TMP would significantly increase the water flux while maintaining a similar salt flux. Consequently, the concentration of salt in the permeate would decrease, leading to an improvement in salt rejection. Furthermore, it is essential to note that the formulations used in commercial membrane fabrication extend beyond the use of MPD and TMC. Commercial membranes often incorporate additives like surfactants to enhance their performance. Thus, with the hollow fiber membrane support developed in this study, further improvements in membrane performance are achievable by improving the reaction conditions.

## 4. Conclusions

Reproducible interfacially polymerized (IP) layers form successfully on polyamide-imide (PAI) hollow fiber membranes with an intermediate polyethyleneimine (PEI) lumen layer. The PAI/PEI support membranes evolve in a single-step fabrication method.

The intermediate PEI lumen layer proves to facilitate a subsequent coating by interfacial polymerization (IP) of MPD and TMC with a high success rate. The resulting composite hollow fiber membranes show reverse osmosis performance with NaCl rejections of around 95% and water permeability of about 1 LMH/bar. We further conclude that during the IP, the reaction between TMC and PEI hinders the transport of MPD to the surface. By increasing the concentration of TMC, this reaction becomes more pronounced as possibly a more prominent PEI-TMC layer forms, resulting in overall lower permeabilities.

Employing an intermediate polyelectrolyte layer proved to facilitate further coating of hollow fiber membranes could potentially become the solution for robust composite hollow fiber membrane fabrication. By varying the coating chemistry and further optimizing the coating solution compositions, we envision developing robust reverse osmosis, nanofiltration, forward osmosis, and organic solvent nanofiltration hollow fiber membranes.

## Figures and Tables

**Table 1:** Bore composition used in the spinning processes described in this work.

Bore Solution	H${}_{2}$O [wt%]	NMP [wt%]	PEI 25k [wt%]	PEI 750k [wt%]
B1	50	50	-	-
B2	45	50	5	-
B3	45	50	-	5

**Table 2:** Spinning parameters of the three fiber batches. The abbreviation “Amb.” refers to ambient temperature.

Batch -	Bore Type -	Temperature Dope Solution [${}^{\circ }$C]	Flow Rate Dope Solution [mL min${}^{-1}$]	Flow Rate Bore [mL min${}^{-1}$]	Take-up Speed [m min${}^{-1}$]	Height [cm]	Air Gap Rel. Humidity [%]	Temp [${}^{\circ }$C]
Unmodified	B1	Amb.	4.5	3.3	2.4	11	65	22
25k	B2	Amb.	4.5	3.4	2.6	11	56	21
750k	B3	Amb.	4.5	3.3	2.5	11	65	22

**Table 3:** Dimensions, porosities, and burst pressures measured for the fibers spun, *n* = 3. The dimensions are determined by the dimension tool for the SEM.

	Unmodified	25k	750k
ID [mm]	0.73 ± 0.01	0.85 ± 0.01	0.73 ± 0.01
Wall thickness [mm]	0.15 ± 0.01	0.14 ± 0.01	0.13 ± 0.01
Porosity [%]	77.1 ± 0.3	79.7 ± 0.2	73.9 ± 0.1
Burst pressure [bar]	13.9 ± 0.6	13.6 ± 0.4	14.0 ± 2
PWP [LMH/bar]	506 ± 69	415 ± 44	366 ± 65

## Data Availability

The data presented in this study are available on request from the corresponding author.

## Supplementary Materials

The following supporting information can be downloaded at: https://www.mdpi.com/article/10.3390/membranes13080741/s1, Figure S1: 3D representation of AFM micrographs of the lumen surface of TFC hollow fiber membranes prepared with 25k supports and different concentrations of MPD and TMC. (a) 1.5wt% MPD and 0.1wt% TMC. (b) 2.5wt% MPD and 0.1wt% TMC. (c) 3.5wt% MPD and 0.1wt% TMC.

## Abbreviations

The following abbreviations are used in this manuscript:

RO	Reverse osmosis
NF	Nanofiltration
FO	Forward osmosis
IP	Interfacial polymerization
TFC	Thin-film composite
PA	Polyamide
MPD	m-phenylenediamine
TMC	Trimesoyl chlorid
PEI	Polyethylenimine
PAI	Polyamide-imide
NMP	1-Methyl-2-pyrrolidinone
EG	Ethylene glycol
TMP	Transmembrane pressure
