# Supplementary material for: Incorporation of an Intermediate Polyelectrolyte Layer for Improved Interfacial Polymerization on PAI Hollow Fiber Membranes
Source: Membranes (Basel). 2023 Aug 18;13(8):741. doi: 10.3390/membranes13080741 (PMC10456695; doi:10.3390/membranes13080741)
Supplement: Supplementary file 1 [file membranes-13-00741-s001.zip › membranes-2435657-supplementary.pdf]

Supplementary Information

Incorporation of an intermediate polyelectrolyte layer for improved interfacial polymerization on PAI hollow fiber membranes

1. Atomic force microscopy

For the evaluation of the surface roughness, atomic force microscopy (AFM) was used to capture 3D surface images of a 3.5x3.5µm area. The AFM was operated under tapping mode in air using a Bruker Dimension Icon with a PPP-NCLR tip from Nanosensors. By analyzing the height profile of the AFM image, the average surface roughness (Ra) of the membrane surface was determined.

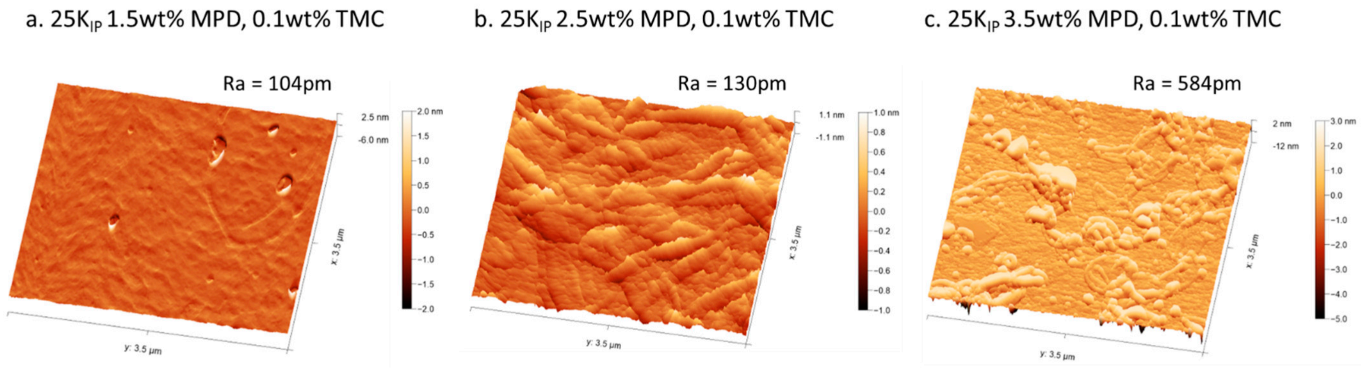

Figure S1. 3D representation of AFM micrographs of the lumen surface of TFC hollow fiber membranes prepared with 25k supports and different concentrations of MPD and TMC. (a.) 1.5wt% MPD and 0.1wt% TMC. (b.) 2.5wt% MPD and 0.1wt% TMC. (c.) 3.5wt% MPD and 0.1wt% TMC.
